# DNA supercoiling differences in bacteria result from disparate DNA gyrase activation by polyamines

**DOI:** 10.1371/journal.pgen.1009085

**Published:** 2020-10-30

**Authors:** Alexandre Duprey, Eduardo A. Groisman

**Affiliations:** 1 Department of Microbial Pathogenesis, Yale School of Medicine, New Haven, CT, United States of America; 2 Yale Microbial Sciences Institute, West Haven, CT, United States of America; Uppsala University, SWEDEN

## Abstract

DNA supercoiling is essential for all living cells because it controls all processes involving DNA. In bacteria, global DNA supercoiling results from the opposing activities of topoisomerase I, which relaxes DNA, and DNA gyrase, which compacts DNA. These enzymes are widely conserved, sharing >91% amino acid identity between the closely related species *Escherichia coli* and *Salmonella enterica* serovar Typhimurium. Why, then, do *E*. *coli* and *Salmonella* exhibit different DNA supercoiling when experiencing the same conditions? We now report that this surprising difference reflects disparate activation of their DNA gyrases by the polyamine spermidine and its precursor putrescine. *In vitro*, *Salmonella* DNA gyrase activity was sensitive to changes in putrescine concentration within the physiological range, whereas activity of the *E*. *coli* enzyme was not. *In vivo*, putrescine activated the *Salmonella* DNA gyrase and spermidine the *E*. *coli* enzyme. High extracellular Mg^2+^ decreased DNA supercoiling exclusively in *Salmonella* by reducing the putrescine concentration. Our results establish the basis for the differences in global DNA supercoiling between *E*. *coli* and *Salmonella*, define a signal transduction pathway regulating DNA supercoiling, and identify potential targets for antibacterial agents.

## Author summary

Living organisms are capable of altering the structure of their DNA in a process called DNA supercoiling. The amount of global DNA supercoiling in bacteria changes depending on environmental and metabolic conditions. Even closely related bacterial species differ in global DNA supercoiling and how it is regulated. Here, we elucidate a mechanism responsible for the discrepancies in global DNA supercoiling between *E*. *coli* and *S*. Typhimurium. We determine that different polyamines regulate *in vivo* DNA supercoiling in the two organisms: spermidine in *E*. *coli* and putrescine in *S*. Typhimurium. In addition, we show that only *S*. Typhimurium alters global DNA supercoiling in response to changes in extracellular Mg^2+^ concentration, and that it does so by modifying putrescine abundance. Our results indicate that differences in the control of shared metabolic pathways can give rise to differences in global DNA supercoiling between closely related bacterial species. Moreover, they suggest ways to inhibit DNA supercoiling in pathogens without impacting commensals or humans.

## Introduction

All living cells supercoil their DNA. The resulting changes in DNA structure enable compaction of the DNA so that it fits within the limited space of a bacterial cell or eukaryotic organelle [1]. DNA supercoiling is necessary for transcription in prokaryotes [2,3] and eukaryotes [4], recombination in bacteriophages [5,6] and eukaryotic viruses, including HIV [7], and chromosome segregation [8]. Because some of the enzymes governing bacterial DNA supercoiling are essential, their pharmacological inhibition is an efficient antibacterial strategy [9,10].

Global DNA supercoiling in bacteria results from the opposing activities of topoisomerase I, which relaxes DNA, and DNA gyrase, which compacts DNA. (Although DNA topoisomerase IV also changes DNA supercoiling, its contribution is negligible compared to that of DNA gyrase under physiological conditions [11].) The global DNA supercoiling effects of these enzymes are in addition to the primarily local effects of gene transcription and DNA replication [12] and nucleoid structuring proteins [13].

Formation of negative supercoiled DNA by the purified DNA gyrase from *Escherichia coli* requires Mg^2+^, adenosine triphosphate (ATP) and spermidine [14], the most abundant polyamine in bacteria [15]. Mg^2+^ and ATP are required for strand cleavage and strand passage, respectively [16,17], but the specific role that spermidine plays has remained unknown. By contrast, neither ATP nor polyamines are required for activity of the investigated bacterial topoisomerase I [18], and its eukaryotic equivalent does not even require Mg^2+^ [19]. Therefore, topoisomerase I activity is currently considered to be independent of metabolic co-factors [9]. Synthesis of topoisomerase I is controlled by a homeostatic mechanism whereby an increase in negatively supercoiled DNA promotes transcription of the topoisomerase I-encoding *topA* gene [20]. It is currently unknown whether specific metabolites control the activities of DNA gyrase and topoisomerase I in living cells.

Polyamines are a family of positively charged molecules consisting of aliphatic hydrocarbon chains harboring several amine groups [21]. Polyamines are found in all living organisms except some Archaea (i.e., Methanobacteriales and Halobacteriales [22]). The three most common polyamines are spermidine, putrescine and spermine. The abundance of individual polyamines often differs across organisms. For example, *E*. *coli* has high amounts of spermidine and its precursor putrescine but lacks the spermidine-derived spermine [15]. Polyamines have RNA- and DNA-binding capacities [23]. In bacteria, they have been implicated in translation [24], protection against acidic [25] and oxidative [26] stresses, iron scavenging [27], and virulence [28,29].

*Salmonella enterica* serovar Typhimurium (*S*. Typhimurium) supercoils its DNA less than *E*. *coli* under basal growth conditions [30]. This is surprising because these two bacterial species are closely related [31] and also because their TopA (topoisomerase I) proteins share 96% amino acid identity, and their GyrA and GyrB proteins, which constitute the two subunits of DNA gyrase, share 91% and 97% identity, respectively. Differences in the latter proteins are responsible, in part, for the distinct global DNA supercoiling behaviors of the two species because: (i) identical mutations in *gyrA* [32] or *gyrB* [33] have different effects on the two species; (ii) the *E*. *coli* DNA gyrase is faster than the *S*. Typhimurium enzyme *in vitro*; and (iii) DNA is less negatively supercoiled in an *E*. *coli* strain expressing a chimeric GyrA protein consisting of the *E*. *coli* GyrA with its 35 C-terminal residues replaced by the corresponding region from the *S*. Typhimurium GyrA than in an *E*. *coli* strain expressing the wild-type *E*. *coli* GyrA [30].

Here, we report that *in vivo*, putrescine and spermidine specifically activate the *E*. *coli* and *S*. Typhimurium DNA gyrases, respectively. We identify an environmental condition that controls DNA supercoiling in *S*. Typhimurium by decreasing putrescine abundance and establish that this condition does not alter DNA supercoiling in *E*. *coli*. Our findings suggest new targets to disrupt DNA supercoiling specifically in bacterial pathogens without harming commensal bacterial species or humans.

## Results

### Spermidine and putrescine stimulate DNA supercoiling in *E*. *coli*

**Fig 1:**
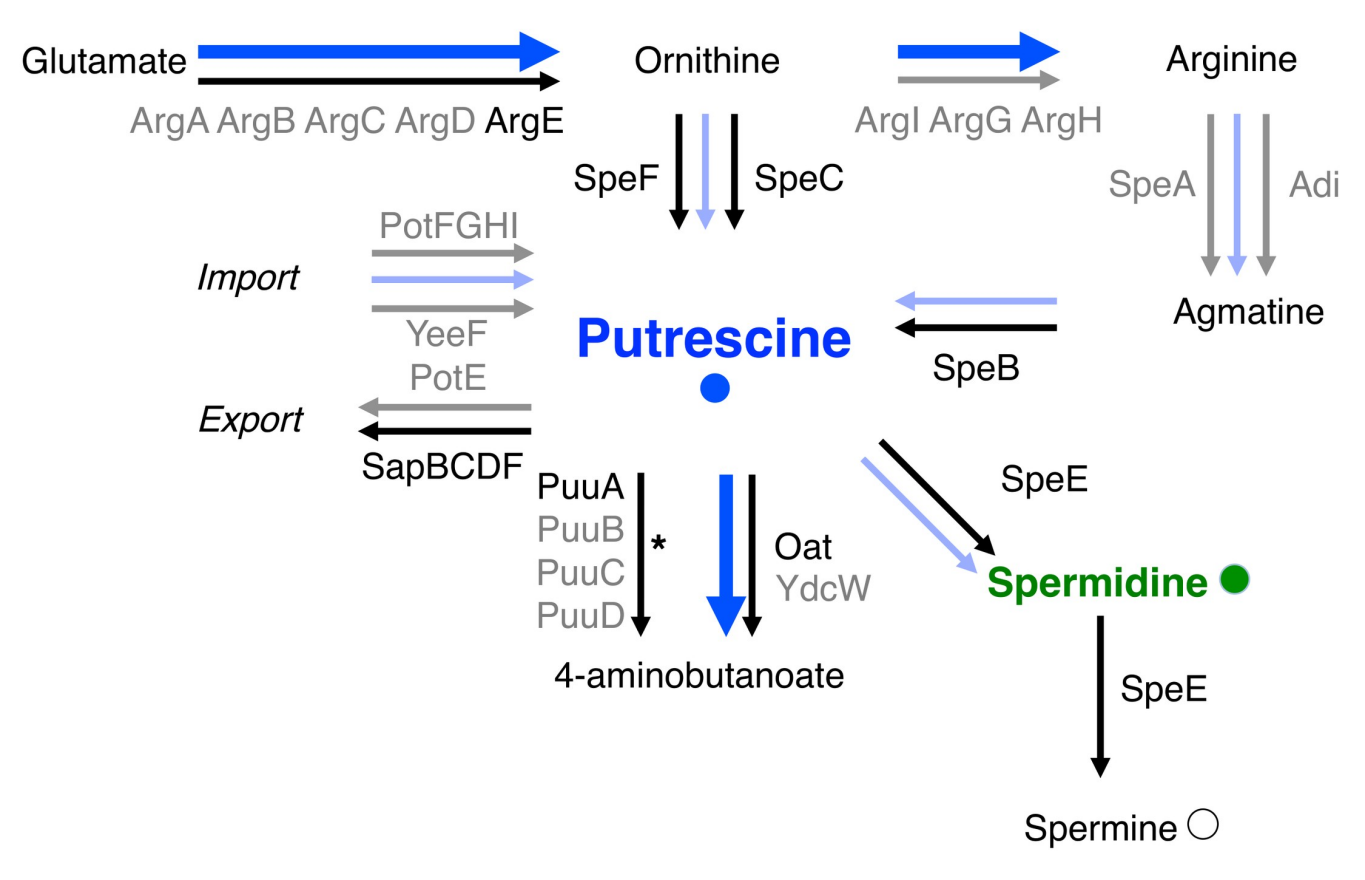
The putrescine biosynthetic and degradative pathways in *S*. Typhimurium and *E*. *coli*. Black and gray arrows represent biochemical reactions carried out by the protein(s) indicated next to them. Black letters indicate proteins investigated in this work, gray letters indicate other proteins related to putrescine abundance. Some intermediate metabolites are omitted for clarity. Blue arrows denote changes in the expression of genes mediating putrescine import, export, biosynthesis or degradation resulting from growth of *S*. Typhimurium in different Mg^2+^ concentrations as measured by the RNA-seq experiment discussed below (Table 1). Bold blue arrows denote pathways upregulated during growth in 10 mM Mg^2+^ compared to 0.8 mM Mg^2+^. Slim light blue arrows denote pathways downregulated during growth in 10 mM Mg^2+^ compared to 0.8 mM Mg^2+^. *: the PuuABCD pathway is present in *E*. *coli* but not in *S*. Typhimurium.

Because activation of the *E*. *coli* DNA gyrase *in vitro* requires spermidine [14], we wondered whether spermidine plays such a role *in vivo*. Thus, we examined the DNA supercoiling status of a reporter plasmid in isogenic wild-type and *speE E*. *coli* using the classical agarose/chloroquine gel method [34]. The *speE* gene specifies a protein that converts putrescine into spermidine, and spermidine into spermine [35] (Fig 1). Therefore, a *speE* null mutant is unable to make spermidine and spermine (Fig 1).

**Table 1:** Extracellular Mg^2+^ controls the mRNA amounts of genes governing putrescine abundance in *S*. *enterica*.

Gene	Expression (10 mM)	Expression (0.8 mM)	Fold change (log2)	q
Arginine biosynthesis				
***argA***	**1422**	**256**	**-2.48**	**<0.001**
***argB***	**2500**	**739**	**-1.76**	**<0.001**
***argC***	**2154**	**446**	**-2.27**	**<0.001**
***argD_2***	**1990**	**521**	**-1.93**	**<0.001**
***argE***	**789**	**355**	**-1.15**	**<0.001**
***argI***	**5296**	**454**	**-3.54**	**<0.001**
***argG***	**3559**	**1825**	**-0.96**	**<0.001**
***argH***	**2533**	**894**	**-1.50**	**<0.001**
Putrescine biosynthesis				
***speA***	**533**	**850**	**0.67**	**0.003**
*adi*	13	11	-0.27	0.35
***speB***	**246**	**487**	**0.99**	**<0.001**
***speC***	**42**	**77**	**0.89**	**<0.001**
*speF*	10	8	-0.22	0.59
Putrescine import				
*potF*	498	548	0.13	0.73
*potG*	224	311	0.47	0.053
*potH*	83	118	0.51	0.17
*potI*	114	154	0.42	0.25
*yeeF* (*plaP*)*	**450**	**1210**	**1.42**	**<0.001**
Putrescine export				
*sapB*	60	70	0.22	0.85
*sapC*	69	78	0.17	0.90
*sapD*	101	126	0.31	0.64
*sapF*	73	92	0.33	0.21
*potE*	7	6	-0.16	0.85
Putrescine degradation				
***speE***	**238**	**662**	**1.47**	**<0.001**
*oat* (*patA*)*	**225**	**84**	**-1.42**	**<0.001**
*ydcW* (*patD*)*	71	61	-0.21	0.51
DNA topoisomerases				
topA	289	298	0.04	0.94
**gyrA**	**377**	**674**	**0.84**	**<0.001**
gyrB	364	499	0.45	0.081
topB	122	160	0.39	0.13
**parC**	**126**	**186**	**0.56**	**0.012**
parE	295	383	0.38	0.15

Differentially expressed genes are indicated in bold face.

Gene expression is indicated in FPKM (Fragments per Kilobase per Million)

*: Name of the corresponding gene in *E*. *coli* MG1655 is given in parentheses when it differs from the name in *S*. Typhimurium strain 14028s.

**Fig 2:**
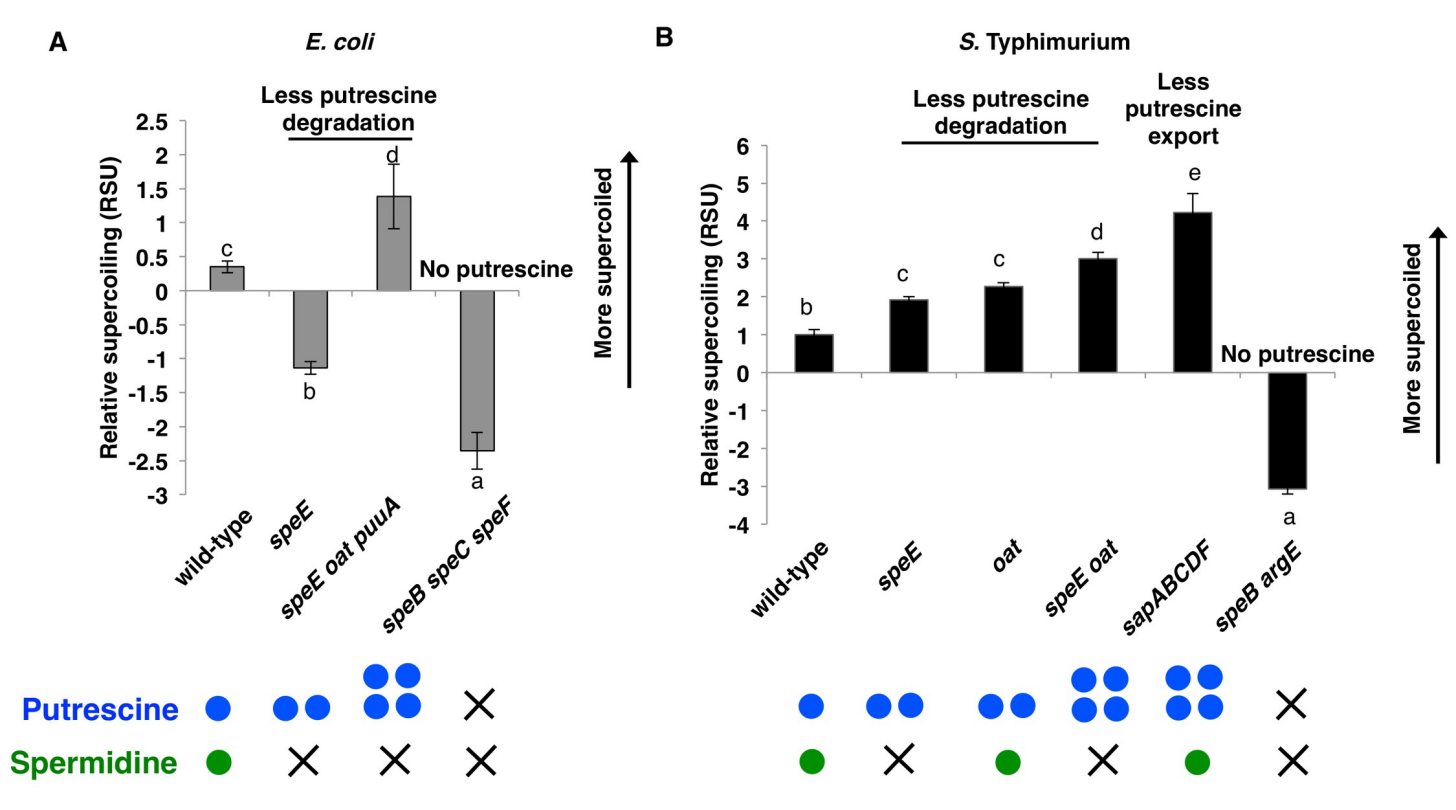
Putrescine increases negative DNA supercoiling *in vivo*. DNA supercoiling in the indicated *E*. *coli* (A) and *S*. Typhimurium (B) strains, which differ in putrescine abundance. *E*. *coli* strains used were: MG1655 (wild-type), AAD85 (*speE*), AAD96 (*speE oat puuA*), AAD95 (*speB speC speF*). *S*. Typhimurium strains used were: 14028s (wild-type), AAD46 (*speE*), JY979 (*oat*), AAD58 (*speE oat*), EG6501 (*sapABCDF*), AAD212 (*speB argE*). Coloured circles qualitatively represent expected concentrations of putrescine (blue) or spermidine (green). Different lowercase letters indicate pairwise statistical significance (Tukey’s HSD, p<0.05, n = 7 for *S*. Typhimurium wild type, n = 4 for *S*. Typhimurium *speE*, n = 2 otherwise). The growth curves corresponding to the cultures used in these experiments are presented in S1 Fig.

**Fig 3:**
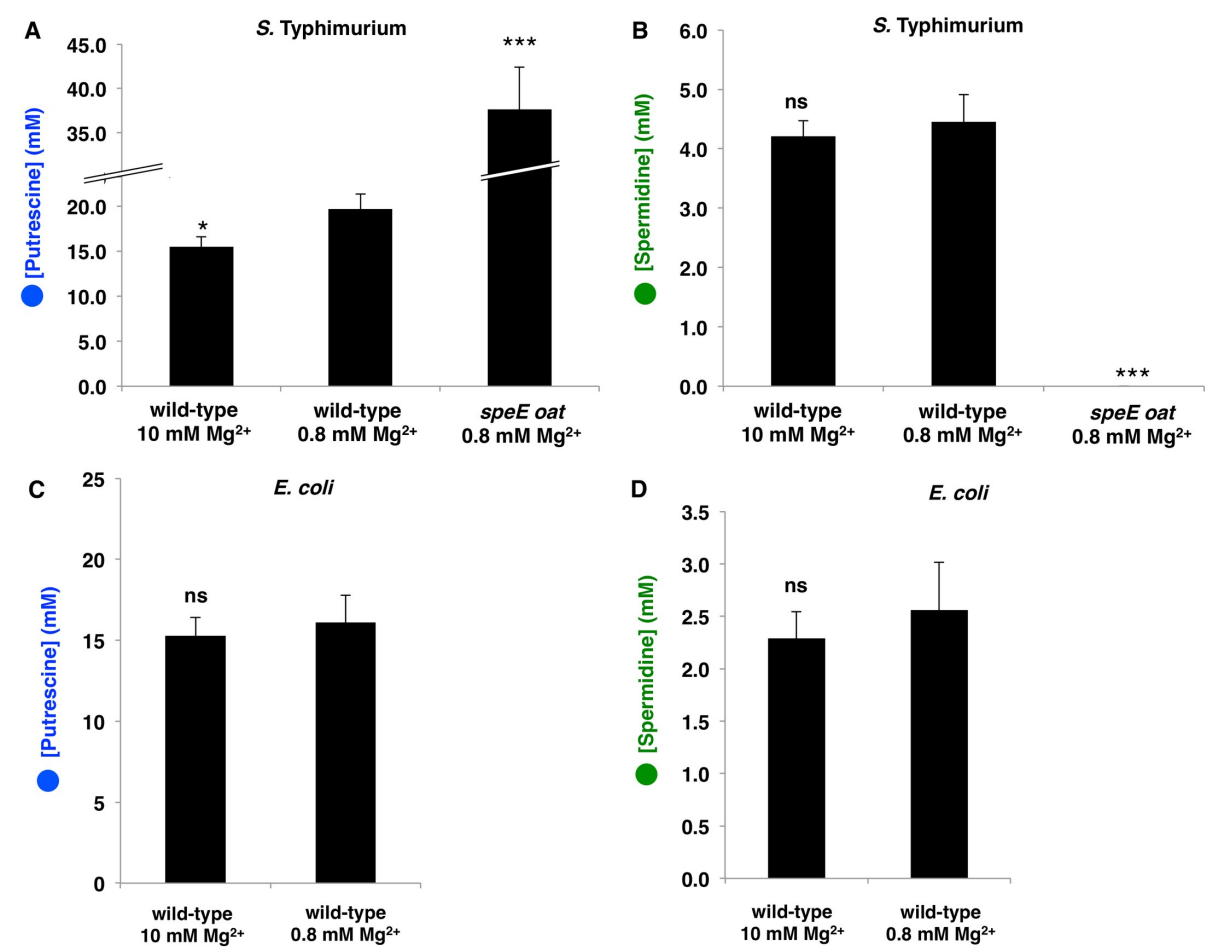
Intracellular concentrations of polyamines determined. A,B: Concentrations of putrescine (A), spermidine (B) and spermine were assessed by HPLC in exponentially growing wild-type *S* Typhimurium strain 14028s or the isogenic *speE oat* mutant (AAD58) in N-minimal medium with the indicated Mg^2+^ concentrations. Stars denote statistical significance compared to wild-type in 800 μM Mg^2+^. ***: p<0.001; *: p<0.05; ns: not significant (Student’s t-test, n = 3). C,D: Same as above but for wild-type *E*. *coli* strain MG1655. None of the tested polyamines was detectable in the *speB speC speF* mutant (AAD95). Spermine was not detected under the investigated conditions (detection limit = 0.2 mM).

DNA was more negatively supercoiled in wild-type *E*. *coli* than in the isogenic *speE* mutant (Fig 2A), suggesting that spermidine and/or spermine increases negative DNA supercoiling. We ascribe this activity to spermidine because spermine was not detected under conditions in which spermidine and putrescine were (Fig 3), in agreement with published results [15].

The *oat* and *puuA* genes specify proteins that convert putrescine into 4-aminobutanoate (Fig 1). Therefore, the *oat puuA speE* triple mutant is predicted to accumulate putrescine but lack spermidine and spermine. DNA was more negatively supercoiled in the *oat puuA speE* triple mutant than in the *speE* single mutant or in wild-type *E*. *coli* (Fig 2A), suggesting that putrescine can increase global DNA supercoiling *in vivo*.

Putrescine is produced from ornithine by either the SpeF or SpeC proteins, depending on the environmental condition, notably extracellular pH [36], or from agmatine by the SpeB protein (Fig 1). The *speB speC speF* triple mutant displayed the lowest negative DNA supercoiling of the investigated *E*. *coli* strains (Fig 2A) as it is unable to make putrescine, spermidine and spermine (Fig 1). Cumulatively, the results in this section indicate that both putrescine and spermidine promote DNA supercoiling in *E*. *coli*, and that spermidine plays a dominant role in this process.

### Putrescine stimulates DNA supercoiling in *S*. Typhimurium, but spermidine has a negligible effect

The SpeE proteins of *S*. Typhimurium and *E*. *coli* share 94% amino acid identity, which is higher than the 90% median amino identity between proteins present in the two species [37]. Therefore, inactivation of the *speE* gene was anticipated to result in analogous phenotypes in *S*. Typhimurium and *E*. *coli*: decreased negative DNA supercoiling. Unexpectedly, DNA was actually more negatively supercoiled in the *S*. Typhimurium *speE* mutant than in the wild-type parent (Fig 2B). This is the exact opposite phenotype observed in *E*. *coli* upon *speE* inactivation (Fig 2A). These results suggest that putrescine is more important for DNA supercoiling in *S*. Typhimurium than in *E*. *coli*.

That putrescine promotes DNA supercoiling in *S*. Typhimurium is further supported by the phenotypes of mutants in pathways predicted to alter putrescine abundance. For instance, DNA was more negatively supercoiled in the *oat speE* double mutant than in *oat* or *speE* single mutants. In turn, DNA was more negatively supercoiled in the latter two mutants than in wild-type *S*. Typhimurium (Fig 2B). Deletion of the putrescine exporter-encoding *sapABCDF* operon [38] increased negative DNA supercoiling more than deletion of the *oat* and *speE* genes (Fig 2A). By contrast, DNA was less negatively supercoiled in a *speB argE* double mutant than in wild-type *S*. Typhimurium (Fig 2B), likely due to the inability of the mutant to synthesize putrescine and spermidine (Fig 1).

Because the *S*. Typhimurium *speB speC speF* triple mutant had a severe growth defect in minimal media, it was not possible to determine its DNA supercoiling status. This is in contrast to the *E*. *coli speB speC speF* triple mutant, which grew well enough to measure DNA supercoiling, albeit not as well as wild-type *E*. *coli*. Therefore, mutations in homologous genes that compromise putrescine abundance result in different phenotypes in *S*. Typhimurium and *E*. *coli*.

The results in this section indicate that putrescine strongly stimulates DNA supercoiling in *S*. Typhimurium and that the contribution of spermidine to this process is minimal. Moreover, they raise the possibility that the different basal global DNA supercoiling status of *S*. Typhimurium and *E*. *coli* [39] result from disparate abundances of specific polyamines in the two species, and/or from the response of their respective topoisomerases or DNA gyrases to these polyamines. As stated above, differences in DNA gyrase activity contribute to the distinct basal DNA supercoiling of *S*. Typhimurium and *E*. *coli* [30].

### The concentrations of putrescine, spermidine, and spermine are similar in *S*. Typhimurium and *E*. *coli*

We determined that wild-type *S*. Typhimurium strain 14028s harbors 20 mM putrescine, 4.5 mM spermidine, and no detectable spermine when grown in N-minimal media with glycerol as carbon source and 0.8 mM Mg^2+^ (Fig 3), a concentration similar to that of free cytoplasmic Mg^2+^ [40]. The abundance of these polyamines is in contrast to those reported for a different *S*. Typhimurium strain grown in complex LB medium in which putrescine and spermine were detected but spermidine was not [41].

Wild-type *E*. *coli* strain MG1655 has polyamine concentrations similar to those of *S*. Typhimurium (15 mM putrescine, 2.5 mM spermidine, no detectable spermine) when grown in the same medium (Fig 3). The latter concentrations are lower than those reported for the *E*. *coli* strain W3110 grown in nutrient broth, albeit in the same order of magnitude [23,42].

The *speE oat* double mutant had more total putrescine (38 mM) than wild-type *S*. Typhimurium (20 mM) and no detectable spermidine (Fig 3). This result makes physiological sense given that the *speE oat* mutant is defective in both putrescine degradation and spermidine synthesis (Fig 1).

Cumulatively, the data in this section argue against the possibility of the disparate DNA supercoiling phenotypes exhibited by *S*. Typhimurium and *E*. *coli* upon *speE* inactivation resulting from differences in polyamine abundance between the two species (Fig 2). Moreover, they suggest that the differences in basal negative DNA supercoiling between *S*. Typhimurium and *E*. *coli* result from the response of their respective DNA gyrases and/or topoisomerases to particular polyamines.

### The *S*. Typhimurium and *E*. *coli* DNA gyrases are differentially activated by individual polyamines

The genetic data presented above argue that negative DNA supercoiling is stimulated by putrescine in *S*. Typhimurium and by spermidine in *E*. *coli* (Fig 2). Thus, we examined the ability of these polyamines to activate the purified DNA gyrases from the two species. To guide the design of an *in vitro* DNA gyrase activation assay using polyamine concentrations in the physiological range, we considered the affinity constants between polyamines and various cellular components and determined that only ~40% of putrescine and ~5% of spermidine are free in the cell [23,42] (i.e., not bound to DNA, RNA, ATP or phospholipids). If the proportion of free polyamines is the same in *S*. Typhimurium and *E*. *coli*, then, based on our measurements of total polyamines (Fig 3), the concentrations of free putrescine and spermidine are estimated to be ~8 mM and ~0.2 mM, respectively.

**Fig 4:**
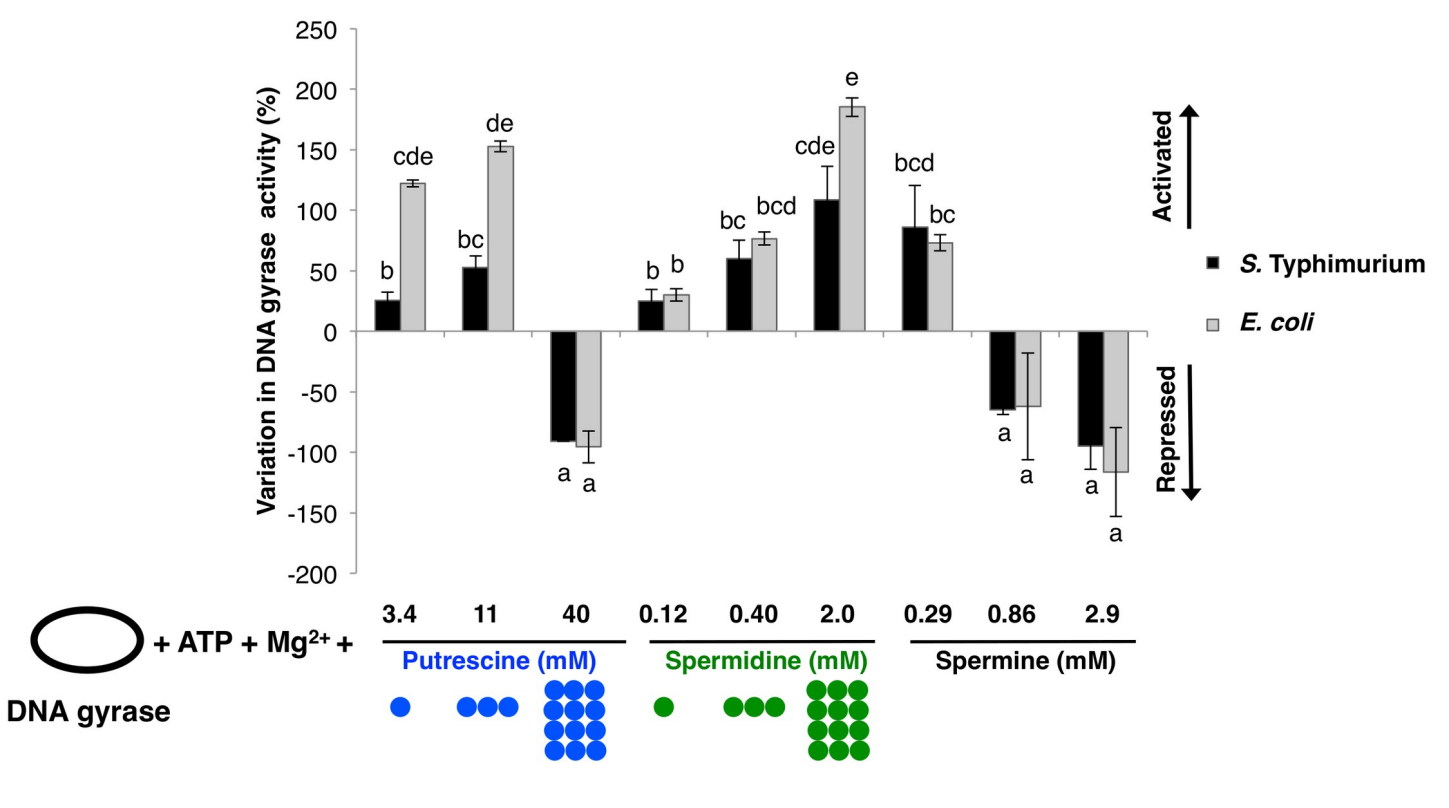
Polyamines regulate the *in vitro* activity of the purified DNA gyrases from *S*. Typhimurium and *E*. *coli*. Topoisomerase-treated plasmid DNA was incubated with purified DNA gyrase alone (reference for 0% variation in DNA gyrase activity) and with the indicated amounts of each polyamine. The experiment was performed with the DNA gyrases from *E*. *coli* and (separately) from *S*. Typhimurium. DNA gyrase activity was calculated as ΔLk/hour from agarose/chloroquine gels and normalized to the activity of the purified DNA gyrase without polyamines. Coloured circles quantitatively represent added concentrations of putrescine (blue) or spermidine (green). Lowercase letters indicate pairwise statistical significance (Tukey’s HSD, p<0.05, n = 2).

The *in vitro* activities of both the *S*. Typhimurium and *E*. *coli* DNA gyrases were stimulated by low concentrations of both putrescine and spermidine (Fig 4). However, the two enzymes responded differently to an increase in the concentrations of these polyamines. For instance, 11 mM putrescine activated the *Salmonella* DNA gyrase as much as 0.4 mM spermidine (Fig 4). Both putrescine and spermidine activated the *E*. *coli* enzyme more strongly than the *S*. Typhimurium DNA gyrase, the effect being greater for putrescine than for spermidine (Fig 4). Activation of the *E*. *coli* DNA gyrase plateaued at a putrescine concentration between 3.4 mM and 11 mM (from +122% to +152%, i.e. a 1.2-fold increase). This is in contrast to the *S*. Typhimurium enzyme, the activity of which increased 2.1-fold between these two concentrations (from +26% to +53%).

Putrescine, spermidine, and spermine modified DNA supercoiling in a DNA gyrase-dependent manner because no changes in DNA supercoiling were observed when DNA gyrase was left out of the reaction (S2 Fig), and also because neither putrescine nor spermidine modified the activity of the purified topoisomerase I from *E*. *coli* (S2 Fig).

Putrescine inhibited both DNA gyrases at 40 mM (Fig 4). This *in vitro* result is unlikely to have physiological significance because inactivation of the putrescine-degrading genes *speE* and *oat* increased putrescine concentration only two-fold *in vivo* (Fig 3), and the putrescine concentration would need to increase five-fold to inhibit DNA gyrase activity (i.e., from 8 to 40 mM free putrescine). Likewise, spermine, which is not detected in *Salmonella* (this work) or *E*. *coli* (this work and [15]), inhibited both DNA gyrases at 0.86 mM, but strongly activated them at 0.29 mM (Fig 4). By contrast, spermidine had no inhibitory effect at the highest tested concentration (i.e., 2 mM) (Fig 4).

Cumulatively, the results in this section indicate that both putrescine and spermidine activate the *S*. Typhimurium and *E*. *coli* DNA gyrases at physiological concentrations. However, these enzymes differ in that the *S*. Typhimurium DNA gyrase is further activated (2.1 fold) by increases in putrescine concentrations that have negligible (1.2 fold) effects on the activity of the *E*. *coli* DNA gyrase.

### An increase in the concentration of extracellular Mg^2+^ decreases global DNA supercoiling in *S*. Typhimurium but not in *E*. *coli*

Mg^2+^ is the most abundant divalent cation in living cells [43]. It is necessary for DNA gyrase activity *in vitro* [14] and competes with polyamines for binding to negatively charged molecules such as DNA [44]. These properties raise the possibility of a regulatory connection existing among the Mg^2+^ concentration, polyamine content, and DNA supercoiling. To test this possibility, we explored whether changes in the Mg^2+^ concentration in the growth media modify global DNA supercoiling in *S*. Typhimurium and *E*. *coli*.

**Fig 5:**
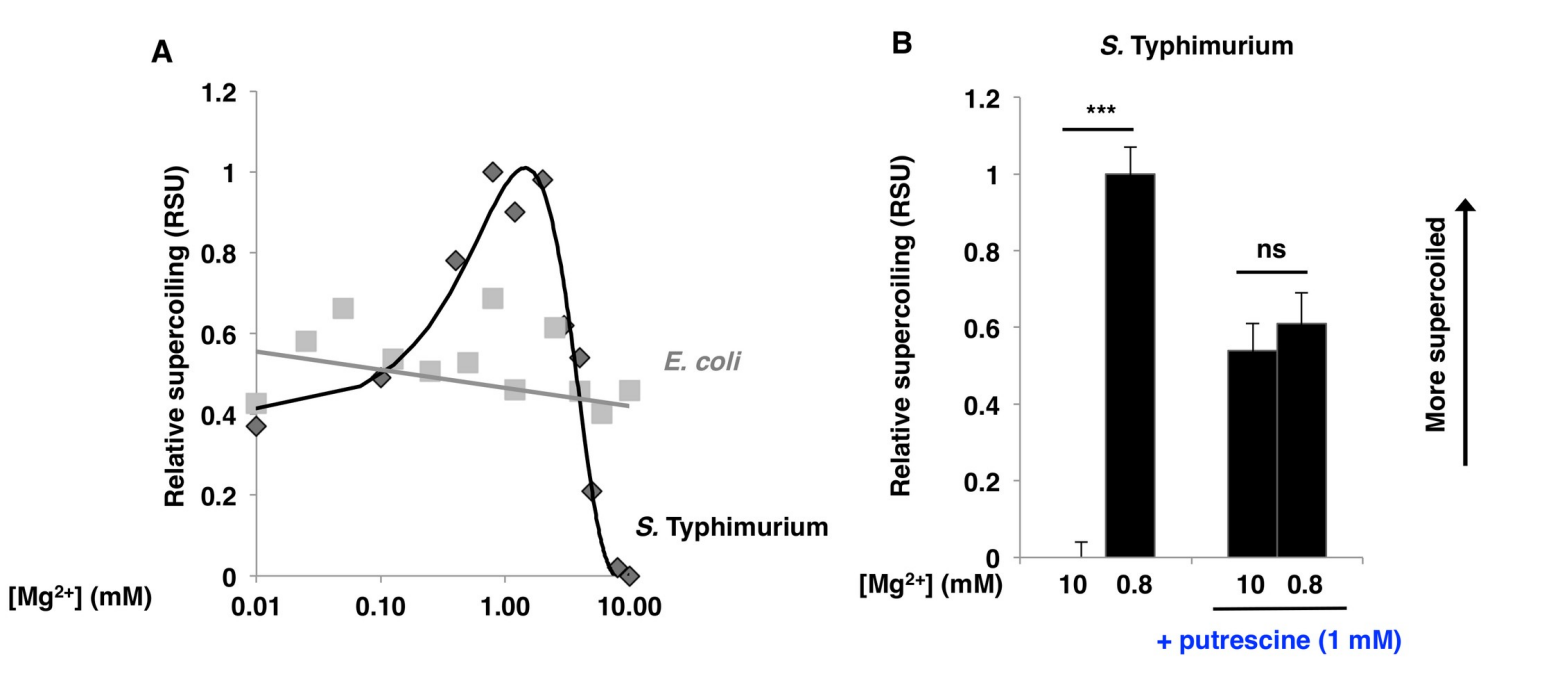
Mg^2+^ controls *S*. Typhimurium DNA supercoiling in a putrescine-dependent manner. A: *In vivo* DNA supercoiling of wild-type *S*. Typhimurium (14028s) (black) and wild-type *E*. *coli* (MG1655) (grey) grown in N-minimal medium with the indicated Mg^2+^ concentrations. B: *In vivo* DNA supercoiling of wild-type *S*. Typhimurium (14028s) grown in N-minimal medium with the indicated Mg^2+^ concentrations in the absence or presence of putrescine (1 mM). ***: p<0.001; ns: not significant (Student’s t-test, n = 2).

DNA supercoiling was maximal in wild-type *S*. Typhimurium experiencing ~1 mM Mg^2+^ (Fig 5A), which corresponds to the concentration of free Mg^2+^ in living cells [40]. DNA became less supercoiled both when the Mg^2+^ concentration in the media decreased to 10 μM Mg^2+^ and when it increased above 2 mM Mg^2+^ (Fig 5A). By contrast, DNA supercoiling in *E*. *coli* was insensitive to changes in the Mg^2+^ concentration in the media between 10 μM and 10 mM (Fig 5A). These results raise the question: How does an increase in Mg^2+^ promote a decrease in DNA supercoiling in *S*. Typhimurium?

### Growth in high Mg^2+^ reduces putrescine abundance in *S*. Typhimurium

Both putrescine (Fig 4) and ATP [45] are required for *in vitro* activity of the *S*. Typhimurium DNA gyrase. Thus, we considered the possibility that growth in high Mg^2+^ decreases DNA supercoiling in *Salmonella* by reducing the concentration of putrescine and/or ATP. Such a reduction would not take place in *E*. *coli* because growth in high Mg^2+^ does not alter DNA supercoiling in this species (Fig 5A).

We determined that the putrescine concentration was 25% higher in *S*. Typhimurium during growth in 0.8 mM than in 10 mM Mg^2+^ (Fig 3). By contrast, the putrescine concentration did not change in *E*. *coli* experiencing these Mg^2+^ concentrations (Fig 3). Putrescine supplementation to the growth medium overcame the decrease in DNA supercoiling provoked by high extracytoplasmic Mg^2+^ in *S*. Typhimurium (Fig 5B). Growth at the high Mg^2+^ concentration resulted in a 1.5-fold decrease in ATP concentration (S3 Fig), which is too small to account for the observed changes in DNA supercoiling [46] (i.e., the decrease in ATP concentration would have to be >5 fold). These results indicate that a growth condition that reduces putrescine abundance also reduces global negative DNA supercoiling in *S*. Typhimurium. How, then, does growth in high Mg^2+^ decrease putrescine abundance?

### Growth in high Mg^2+^ reduces the mRNA amounts of genes that increase putrescine abundance

We carried out an RNA-seq experiment to identify genes differentially expressed at Mg^2+^ concentrations resulting in high versus low global negative DNA supercoiling (0.8 mM and 10 mM, respectively) in wild-type *S*. Typhimurium. Growth in 10 mM Mg^2+^ changed the mRNA abundance of multiple genes that encode proteins predicted to lower putrescine amounts (Fig 1; values for each gene presented in Table **1**). For example, the mRNA abundance of the *speA*, *speB*, and *speC* genes was lower in 10 mM Mg^2+^ than in 0.8 mM Mg^2+^ (Table **1**). *speA* and *speB* specify enzymes that participate in the synthesis of putrescine from arginine (Fig 1); and *speC* mediates putrescine synthesis from ornithine (Fig 1).

The mRNA abundance of the putrescine-degrading *oat* gene and of the *arg* genes mediating the conversion of ornithine into arginine (Fig 1) was higher in 10 mM Mg^2+^ than in 0.8 mM Mg^2+^. The most strongly upregulated gene in 10 mM relative to 0.8 mM Mg^2+^ was *argI*, which specifies the rate-limiting enzyme in the conversion of ornithine into arginine (Fig 1). Thus, the transcriptional response to high Mg^2+^ favors expression of genes that promote putrescine degradation and deplete the putrescine precursor ornithine. However, similar agmatinase activity mediated by the SpeB protein was observed in crude extracts prepared from *S*. Typhimurium grown at 10 mM and 0.8 mM Mg^2+^ (S4 Fig), and ornithine decarboxylase activity mediated by the SpeC and SpeF proteins was undetectable(S4 Fig). Therefore, it is presently unclear which of the genes whose transcription changes in 10 mM vs. 0.8 mM Mg^2+^ (Table **1**) is responsible for the resulting change in putrescine abundance (Fig 3).

The mRNA abundance of the putrescine importer *yeeF* gene [47] was lower in 10 mM relative to 0.8 mM Mg^2+^. Taken together with the data presented above, this result indicates that high Mg^2+^ reduces the putrescine concentration in *S*. Typhimurium by altering the expression of genes predicted to decrease putrescine import and synthesis, and to increase putrescine degradation.

Finally, expression of *gyrA* (but not *gyrB*), which is also significantly regulated by Mg^2+^ in the growth media though not to the extent of *argI*, *speE* or *oat*, may contribute to the observed changes in global negative DNA supercoiling. Therefore, the coordinated response to Mg^2+^ (rather than individual enzymatic activities), appears to be responsible for the observed variations in polyamine amounts (Fig 3) and the resulting changes in global negative DNA supercoiling. The effects of Mg^2+^ point to specific gene regulation by Mg^2+^ rather than effects on DNA supercoiling-sensitive promoters because none of the genes differentially expressed at 10 mM vs. 0.8 mM Mg^2+^ displayed sensitivity to DNA supercoiling when 11 different DNA supercoiling-altering conditions were used to examine their expression behavior (see S5 Fig and GEO entry GSE137586).

## Discussion

We have now established that different polyamines activate the DNA gyrases from *S*. Typhimurium and *E*. *coli*. The different response of these DNA gyrases to the polyamines putrescine and spermidine dictates the distinct basal global DNA supercoiling set point of the two species. We determined that environmental Mg^2+^ regulates DNA supercoiling in *S*. Typhimurium, but not in *E*. *coli*, by altering putrescine abundance. Our findings reveal a novel physiological role for polyamines, which play critical functions in all domains of life [48]. Moreover, they help explain why DNA is less negatively supercoiled in *S*. Typhimurium than in *E*. *coli* when these bacteria experience the same growth condition. And they suggest potential targets for antibacterial agents to inhibit the essential process of DNA supercoiling.

### The molecular basis for the DNA supercoiling differences between *S*. Typhimurium and *E*. *coli*

**Fig 6:**
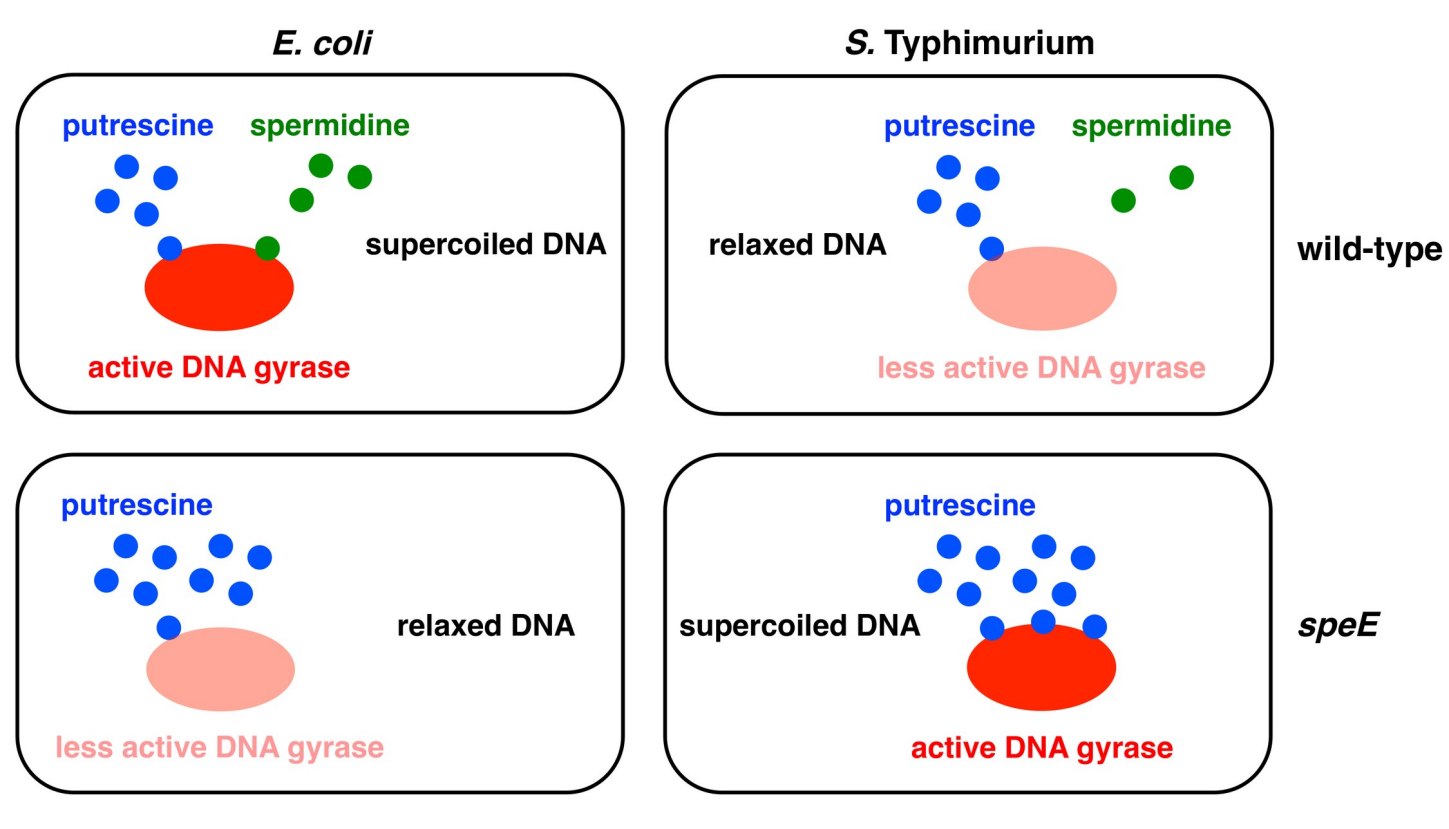
Differential sensitivity of DNA gyrases to distinct polyamines dictates DNA supercoiling differences between *E*. *coli* and *S*. Typhimurium. *E*. *coli* DNA gyrase activity requires minimal amounts of putrescine and is therefore insensitive to variations in putrescine amounts (top left). As a result, the decreased DNA supercoiling resulting from loss of spermidine in a *E*. *coli speE* mutant is not compensated by increased amounts of putrescine, resulting in relaxed DNA (bottom left). *S*. Typhimurium DNA gyrase activity requires high amounts of putrescine to be activated and is highly sensitive to variations in putrescine amounts (top right). Putrescine accumulation in a *S*. Typhimurium *speE* mutant hyperactivates its DNA gyrase resulting in more negatively supercoiled DNA (bottom right).

The activity of the purified DNA gyrases from *S*. Typhimurium and *E*. *coli* is enhanced or inhibited by individual polyamines depending on their concentrations (Fig 4). This result explains the surprising finding that the *S*. Typhimurium *speE* mutant, which is unable to make spermidine but accumulates its precursor putrescine (Fig 1), is more supercoiled than wild-type *S*. Typhimurium (Fig 2B), but that the equivalent *speE* mutation in *E*. *coli* decreases DNA supercoiling below the values of wild-type *E*. *coli* (Fig 2A). Although putrescine activates the *E*. *coli* DNA gyrase at lower concentrations than the *S*. Typhimurium DNA gyrase, further increases in putrescine concentration stimulate *S*. Typhimurium DNA gyrase activity but not that of *E*. *coli* (Fig 4). Therefore, the increased putrescine amounts resulting from *speE* inactivation in *E*. *coli* have little effect on its DNA gyrase because the enzyme is already maximally activated (Fig 6).

By contrast, the putrescine accumulation resulting from *speE* inactivation in *S*. Typhimurium furthers DNA supercoiling because activation of its DNA gyrase requires higher putrescine amounts than does activation of the *E*. *coli* DNA gyrase (Fig 3). That conditions resulting in putrescine accumulation hyperactivate DNA gyrase in *S*. Typhimurium is further supported by the increased DNA supercoiling exhibited by the *sapABCDF* mutant (Fig 2), which is defective in putrescine export [38]. The different DNA supercoiling phenotypes resulting from inactivation of the *speE* gene in *S*. Typhimurium and *E*. *coli* are reminiscent of the different behaviors exhibited by these species harboring equivalent mutations in the *gyrB*, *seqA*, and *mukB* genes [33]

DNA gyrase is a tetramer consisting of two GyrA and two GyrB proteins [9]. The behavior of organisms with chimeric DNA gyrases originating from *S*. Typhimurium and *E*. *coli*, and the clustering of amino acid differences between the two GyrA proteins in the C-terminus, implicate this region in the species-specific DNA supercoiling behavior. Although the *E*. *coli* DNA gyrase is faster *in vitro* than the *S*. Typhimurium DNA gyrase in the presence of spermidine, a *S*. Typhimurium strain bearing the *E*. *coli* GyrA protein exhibited decreased DNA supercoiling compared to wild-type *S*. Typhimurium [30]. Our findings raise the possibility of putrescine and spermidine interacting with the C-terminal region of GyrA to activate the *S*. Typhimurium and *E*. *coli* DNA gyrases, respectively. Under this hypothesis, loss of supercoiling in the *S*. Typhimurium strain with the *E*. *coli* GyrA may reflect disrupted activation by polyamines.

Growth in >2 mM Mg^2+^ reduces DNA supercoiling in *S*. Typhimurium (Fig 5A) by promoting expression of genes specifying products that reduce putrescine accumulation (Table **1**). Because the Mg^2+^ effect on DNA supercoiling is independent of the ATP control of DNA gyrase activity [46], the uncovered mechanism allows *S*. Typhimurium to modify DNA supercoiling while avoiding disruption of energy-dependent cellular processes. Furthermore, it enables *S*. Typhimurium to alter DNA supercoiling in response to different, and *a priori* independent, metabolic signals–ATP and putrescine.

*S*. Typhimurium experiences a decrease in global negative DNA supercoiling inside mammalian cells [49]. A mildly acidic pH was previously proposed to play a role based on an *in vivo* synergistic effect of the DNA gyrase inhibitor novobiocin and acidic pH on DNA relaxation [50]. However, this observation can be explained by a mildly acidic pH increasing permeability to novobiocin [51]. Changes in the abundance of putrescine and spermidine, or in a metabolite yet to be identified, may contribute to the reported decrease in global DNA supercoiling.

*E*. *coli* differs from *S*. Typhimurium in that it does not alter its basal DNA supercoiling in response to an increase in Mg^2+^ concentration in its surroundings (Fig 5A). Under the investigated conditions, the levels of negative DNA supercoiling in *E*. *coli* are similar to those in *S*. Typhimurium (0.5 RSU compared to 0–1 RSU, respectively). This argues against the C-terminus of GyrA being responsible for the reported lower DNA supercoiling in *S*. Typhimurium vs. *E*. *coli* [30]. However, the latter experiments were carried out using both a different *S*. Typhimurium strain (LT2-derived versus 14028s in the current study) and growth conditions (LB at 30°C versus defined media at 37°C in the current study), which raises the possibility of the differences in DNA supercoiling between *S*. Typhimurium and *E*. *coli* resulting from the signals they respond to and the magnitude of those responses. The DNA gyrase subunits of *S*. Typhimurium and *E*. *coli* are >90% identical, and this is also the case for the SpeE protein. Therefore, these findings alongside others [30,52,53] demonstrate how phenotypic differences between closely related bacterial species can result from allelic differences in conserved genes and in their regulation, rather than from the presence of a gene(s) in one species and its absence from another.

### Inhibiting DNA supercoiling by manipulating polyamine abundance

The vast majority of antibacterial agents that inhibit DNA supercoiling do so by targeting DNA gyrase [54]. Unfortunately, many of these agents are toxic to humans [55], and resistance to these agents is spreading quickly [56]. Furthermore, despite continued efforts to discover compounds that alter DNA gyrase activity [57], there has been no clinical success since the year 2000.

The identification of putrescine and spermidine as key regulators of DNA gyrase *in vivo* and *in vitro* raises the possibility of altering DNA supercoiling by targeting the pathways governing polyamine abundance (as opposed to DNA gyrase). That polyamine biosynthesis is essential in several pathogens (reviewed in [48]) supports this possibility. In *S*. Typhimurium, there is a direct correlation between DNA supercoiling and expression of the invasion gene *invA* [58], and DNA supercoiling decreases when *S*. Typhimurium is inside macrophages [59], promoting expression of genes required for intramacrophage survival [59]. Moreover, mutants defective in putrescine synthesis or export exhibit aberrant DNA supercoiling (Fig 2B) and are attenuated for virulence [28,29].

Spermine inhibited DNA gyrase at submicromolar concentrations (Fig 4). This raises the possibility of organisms using spermine as an endogenous DNA gyrase inhibitor. It also suggests using a spermine scaffold to design novel DNA gyrase inhibitors.

Finally, humans lack some of the putrescine biosynthetic enzymes utilized by bacteria (S6 Fig) as well as putrescine transporters, making it less likely that an agent targeting putrescine abundance in bacteria would inhibit human proteins carrying out analogous functions. That pathogenic *S*. Typhimurium and commensal *E*. *coli* differ in their control of DNA supercoiling suggests that an antimicrobial agent may target pathogen-specific pathways while sparing those operating in commensal organisms.

## Materials and methods

### Bacterial strains, plasmids and growth conditions

Strains, plasmids and primers are described in S1 Table.

Mutations were created by λred recombination [60]. Plasmid pSIM6 was used to supply λred in all cases. After PCR verification of the strains, mutations were moved into a clean genetic background using P22-mediated transduction in the case of *S*. Typhimurium [61] and P1-mediated transduction in the case of *E*. *coli*.

Strains AAD35, AAD46 and AAD85 were built by λred recombination using pKD4, pKD3 and pKD4 as templates, respectively, and primer pairs 16608/16609, 16651/16652, 16834/16835, respectively.

Strain AAD58 was built by P22 transduction using a lysate prepared in strain AAD46 to infect strain JY979.

Strain AAD181 was built by P22 transduction using a lysate prepared in strain AAD35 to infect strain AAD58.

For strain AAD212, single *argE*::*kan* and *speB*::*cat* mutations were built in separate strains by λred recombination using pKD4 and pKD3 as templates, respectively, and primer pairs 16698/16699 and 16702/16703, respectively. The *speB*::*cat* strain (AAD62) was then transformed by plasmid pCP20 to remove the *cat* cassette and yield a Δ*speB* strain (AAD65). Finally, the Δ*speB* strain was transduced with a P22 lysate prepared in strain AAD61 (*argE*::*kan*) to yield strain AAD212.

For strain AAD96, single *oat*::*cat* and *puuA*::*cat* mutations were built separately by λred recombination using pKD3 and pKD4 as templates, respectively, and primer pairs 16840/16841 and 16842/16843, respectively. The *oat*::*cat* (AAD86) strain was then transformed with plasmid pCP20 to remove the *cat* cassette and yield Δ*oat* strain (AAD88). Finally, the Δ*oat* strain was sequentially transduced with P1 lysate from AAD85 (*speE*::*kan*) and from AAD91 (*puuA*::*cat*) to yield strain AAD96.

For strain AAD95, single *speB*::*cat*, *speC*::*kan* and *speF*::*cat* mutations were built in separate strains by λred recombination, using pKD3, pKD4 and pKD3 as templates, respectively, and primer pairs 16832/16833, 16838/16839, and 16836/16837 respectively. The *speB*::*cat* strain (AAD89) was then transformed with plasmid pCP20 to remove the *cat* cassette and yield a Δ*speB* strain (AAD92). Finally, the Δ*speB* strain was sequentially transduced with a P1 lysate prepared in the *speC*::*kan* strain (AAD87) and from the *speF*::*cat* strain (AAD90) to yield strain AAD95.

Strains AAD85, AAD95 and AAD96 were sequenced alongside the parental MG1655, and Snippy (https://github.com/tseemann/snippy) was used to detect mutations. AAD96 has a mutation that specifies a WaaO protein with the Asp36Glu substitution. The four strains were otherwise isogenic except for the intended mutations. Data are available in the Sequence Read Archive (SRA) under accession numbers SRR12282603, SRR12282604, SRR12282605, SRR12282606.

All strains were grown in HH800 minimal medium at 37°C except otherwise indicated. HH800 is N-minimal medium[62] supplemented with 0.8 mM MgCl_2_, 0.1% casamino acids, 0.27% glycerol, and adjusted to pH 7.7. HH was the same medium, but with 10 mM MgCl_2_, and was used as a high magnesium concentration to investigate regulation of polyamine abundance by Mg^2+^. Antibiotics were used at the following concentrations: ampicillin, 50μg/mL; kanamycin, 50μg/mL; chloramphenicol, 25μg/mL. *E*. *coli* strains were further supplemented with 1μg/mL biotin and 1μg/mL thiamine when grown in minimal medium.

### Measurement of DNA supercoiling on agarose/chloroquine gels

Overnight precultures of strains bearing plasmid pJV were washed once in water, then diluted into fresh HH800 medium to a starting OD_600_ of 0.05. Cells were grown until mid-exponential phase (OD_600_ = 0.7 ± 0.1 for wild-type, and adjusted accordingly for mutants with altered growth), then plasmids were immediately extracted using a QIAGen Plasmid Mini kit. 800 ng of purified plasmid for each sample was then loaded on a Tris-Borate-EDTA, 0.8% agarose, 2.5 μg/mL chloroquine gel. Gels were run overnight at 1.3 V/cm, washed in water for at least 4 h, then stained using EZ-vision (VWR) and imaged with an ImageQuant LAS400 (GE healthcare). The intensity of each band was quantified with ImageJ. The linking number (Lk) of the top band was arbitrarily set to 0, then the band immediately below had Lk = 1, the next one Lk = 2… The intensity-weighted average Lk was calculated for each lane. The measured DNA supercoiling for *S*. Typhimurium was normalized across experiments to the supercoiling exhibited by the wild-type strain 14028s following growth in HH (= HH800 + 10 mM MgCl_2_), defined as 0 Relative Supercoiling Units (RSU), and the supercoiling in WT in HH800, defined as 1 RSU.

### Considerations about quantification of DNA supercoiling

This work concerns *in vivo* DNA supercoiling, which is overall negative. Therefore, relaxed DNA (i.e. supercoiling closer to 0) is referred to as “low supercoiling” and has low RSU values. By contrast, supercoiled DNA (i.e., strongly negative DNA supercoiling) is referred to “high supercoiling” and has high values in RSU units. Because apparent linking numbers on agarose/chloroquine gels often vary from gel to gel, we use a relative scale instead (RSUs), which allows easy comparison of different gels thanks to a common set of controls. Under the investigated conditions, one linking number difference out of 7 or 8 visible bands typically converts into 2 RSUs. As we focused on quantitative variations in DNA supercoiling rather than absolute values, we did not attempt to distinguish positive and negative DNA supercoiling on gels.

### HPLC measurement of total polyamines

Strains were cultured as described above for the determination of DNA supercoiling. Five OD_600_ units were pelleted and washed three times with PBS. Cells were then resuspended in 200 μL lysis buffer (100 mM MOPS, 50 mM NaCl, 20 mM MgCl_2_, pH 8.0) and plated to obtain Colony Forming Units (CFUs).

Cells were frozen in liquid nitrogen, then thawed at 37°C three times. 60 μL trichloroacetic acid 40% was added and the mixture was incubated on ice for 5 min. The supernatant was submitted for derivatization.

Derivatization was performed by adding 250 μL NaOH 2M, 10 μL benzoyl chloride and incubating at room temperature for 20 min. The reaction was stopped by adding 400 μL NaCl, then polyamines were extracted using 300 μL diethyl ether. The ether was evaporated at 37°C and polyamines were dissolved in methanol/water 45%/55% (v/v).

For the standard curve, a mixture of 20 mM putrescine, 10 mM spermidine and 10 mM spermine was made. This mixture was serially diluted in 4-fold increments. 50 μL of the polyamine mix was added to 200 μL lysis buffer, then derivatized as before.

HPLC was performed on a Waters Acquity UPLC H Class with PDA detector (Waters, Milford, MA). The column was a Waters Acquity UPLC BEH C18, 1.7 μm (Waters 186005604), kept at 60°C throughout the experiment. Flow rate was 0.3 mL/min. Solvent was 45% methanol/55% water (v/v). Detection was performed in UV at 254 nm.

Cell volume was measured in a separate experiment by phase microscopy, and estimated to be 4 μm^3^ for the tested strains and conditions.

Intracellular polyamine concentrations were then calculated from the measured concentrations by HPLC, the CFUs and the cell volume.

### RNA-seq analysis

Overnight precultures of strains bearing plasmid pJV were washed once in water, then diluted into fresh HH800 medium supplemented or not with 10 mM Mg^2+^ to a starting OD_600_ of 0.05. Cells were grown until OD_600_ = 0.8 ± 0.1 (similarly to supercoiling measurements) and RNA was extracted using a QIAGen RNeasy Mini kit. In addition to the included DNase treatment in the kit, DNA was further eliminated by treatment by turbo DNase (Ambion). RNA was finally re-purified using a QIAGen RNeasy Mini kit.

Library construction and sequencing was performed by the Yale Center for Genome Analysis. rRNA was depleted using a RiboZero kit (Illumina). cDNA synthesis was performed by adding A bases to the 3’ end of fragments, followed by oligodT priming. The 4 samples were barcoded and multiplexed into a single flow cell. Sequencing was performed by on an HS4000 (Illumina), Hiseq 75x2 paired-end, unstranded.

Reads were mapped to 14028s genome (Genbank CP001363.1) using bowtie 2.2.9 [63] and differential expression analysis was performed using cuffdiff from the cufflinks 2.2.1 [64] package. Data was deposited to GEO (GSE150757)

### Assay of ornithine decarboxylase and agmatinase activities

Cells were grown until mid-exponential phase as for DNA supercoiling measurements. Then, cells were washed twice in PBS, and sonicated on a Bioruptor sonicator (Diagenode, NJ, USA) with the following settings: High intensity, 30s ON/30s OFF, 15 min. The supernatant was used to determine activities and protein concentrations.

Agmatinase activity was determined by assaying the release of urea by alpha-isonitroso propiophenone [65] and ornithine decarboxylase by solubilization of 2,4,6 trinitrobenzenesulfonic acid adducts into DMSO [66].

Protein concentrations were determined using the Bradford colorimetric assay.

### Examining the activities of topoisomerase I and DNA gyrase

Wild-type *S*. Typhimurium strain14028s transformed with pJV was grown in HH800 + 10 mM MgCl_2_ + 25 μg/mL novobiocin until OD_600_ = 1, then pJV was extracted from 400 mL of culture using a QIAGen Plasmid Maxi kit. This yielded a moderately relaxed pJV.

For topoisomerase I assays, 800 ng of purified pJV was incubated with 0.5 units of DNA topoisomerase I (NEB M0301) in Cutsmart buffer (NEB B7204) at 37°C for 2 h, then inactivated at 65°C for 20 min.

For gyrase assays pJV was first further relaxed by topoisomerase I as for the topoisomerase I assay. Then, for *E*. *coli* gyrase assays, 800 ng of relaxed pJV was incubated with 2 units of DNA gyrase (TopoGen TG-2000G-3) at 37°C for 5 h, then inactivated at 65°C for 20 min. For *S*. Typhimurium gyrase assays, 800 ng of relaxed pJV was incubated with 0.62 pmol GyrA and 0.62 pmol GyrB (both purified from SL1344, which has 100% identity with 14028s gyrase subunits, gifted by Lesley Mitchenall and Anthony Maxwell, John Innes Centre, Norwich, UK, [32]) at 37°C for 90 min, then inactivated at 65°C for 20 min. The reaction buffer was as follows: Tris pH 7.5, 35 mM; KCl, 24 mM; MgCl_2_, 4 mM; DTT, 2 mM; ATP, 1 mM; glycerol, 6.5% (v/v); Bovine Serum Albumin, 0.1 mg/mL.

In all cases, the whole reaction was loaded onto a Tris-Borate-EDTA, 0.8% agarose gel, 0.4 μg/mL chloroquine gel. Gels were migrated, stained, imaged and quantified as for *in vivo* DNA supercoiling gels. DNA gyrase activity was defined as the increase in average Lk, per hour, compared to the initial topoisomer distribution. DNA gyrase activity in the absence of any polyamine was used as a baseline (i.e. 0% relative variation).

## Supporting information

S1 Fig Growth curves for the various strains presented in Fig 2. A: *E*. *coli* strains B: *S*. Typhimurium strains. Each line represents a single biological replicate. The large red square represents the point where samples were taken for DNA supercoiling.
(PDF)

S2 Fig *In vitro* effect of putrescine, spermidine and ornithine on topoisomerase I. Assays were conducted as described in Methods using the indicated amounts of the listed metabolites.
(PDF)

S3 Fig ATP amounts vary little in response to excess Mg^2+^. Intracellular ATP concentrations of wild-type S. Typhimurium (14028s) were measured using the Bac-Titer Glo assay. *: p<0.05 (Student’s t-test, n = 3)
(PDF)

S4 Fig Activity of putrescine biosynthetic enzymes in crude S. Typhimurium extracts. **Agmatinase and ornithine decarboxylase activities were assayed in crude extracts** from wild-type *S*. Typhimurium **(14028s).** ns: not significant (Student’s t-test, n = 3)
(PDF)

S5 Fig Expression of key putrescine-related genes is not dependent on DNA supercoiling. Expression of the presented *S*. Typhimurium genes (in FPKM) was plotted as a function of DNA supercoiling (data taken from GEO entry GSE137586). Open circles represent independent data points, and lines represent linear regressions.
(PDF)

S6 Fig The putrescine biosynthetic and degradative pathways in *H*. *sapiens*. Note that OAT is unrelated to the bacterial Oat and catalyzes a different reaction.
(PDF)

S1 Table Strains, plasmids and primers used in this study. (PDF)
