# Supplementary material for: DNA supercoiling differences in bacteria result from disparate DNA gyrase activation by polyamines
Source: PLoS Genet. 2020 Oct 30;16(10):e1009085. doi: 10.1371/journal.pgen.1009085 (PMC7598504; doi:10.1371/journal.pgen.1009085)
Supplement: S1 Fig — A: E. coli strains B: S. Typhimurium strains. Each line represents a single biological replicate. The large red square represents the point where samples were taken for DNA supercoiling. (PDF) [file pgen.1009085.s001.pdf]

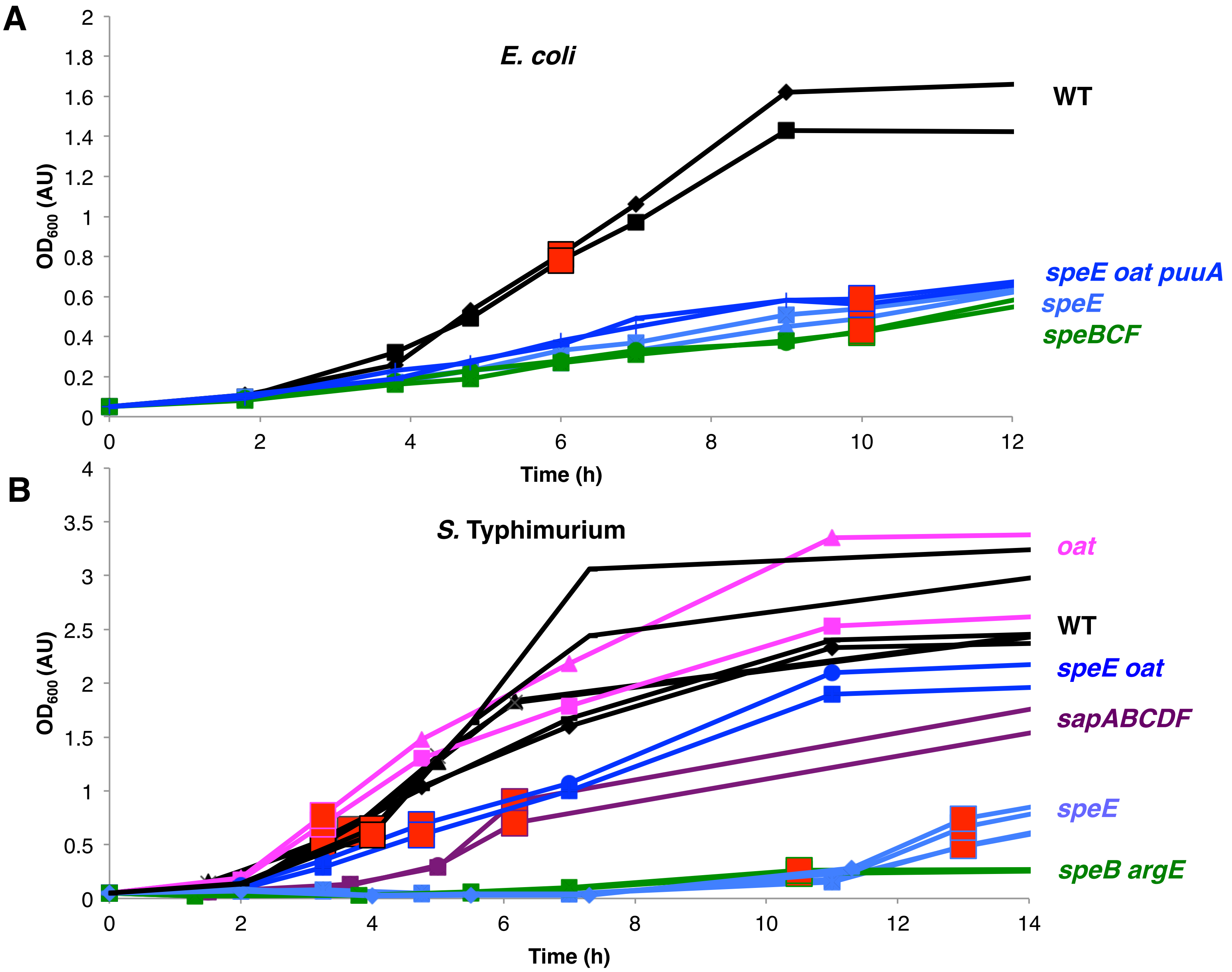

Figure S1: Growth curves for the various strains presented in Figure 2.

A: *E. coli* strains

B: *S.* Typhimurium strains.

Each line represents a single biological replicate. The large red square represents the point where samples were taken for DNA supercoiling
