# Supplementary material for: DNA supercoiling differences in bacteria result from disparate DNA gyrase activation by polyamines
Source: PLoS Genet. 2020 Oct 30;16(10):e1009085. doi: 10.1371/journal.pgen.1009085 (PMC7598504; doi:10.1371/journal.pgen.1009085)
Supplement: S2 Fig — Assays were conducted as described in Methods using the indicated amounts of the listed metabolites. (PDF) [file pgen.1009085.s002.pdf]

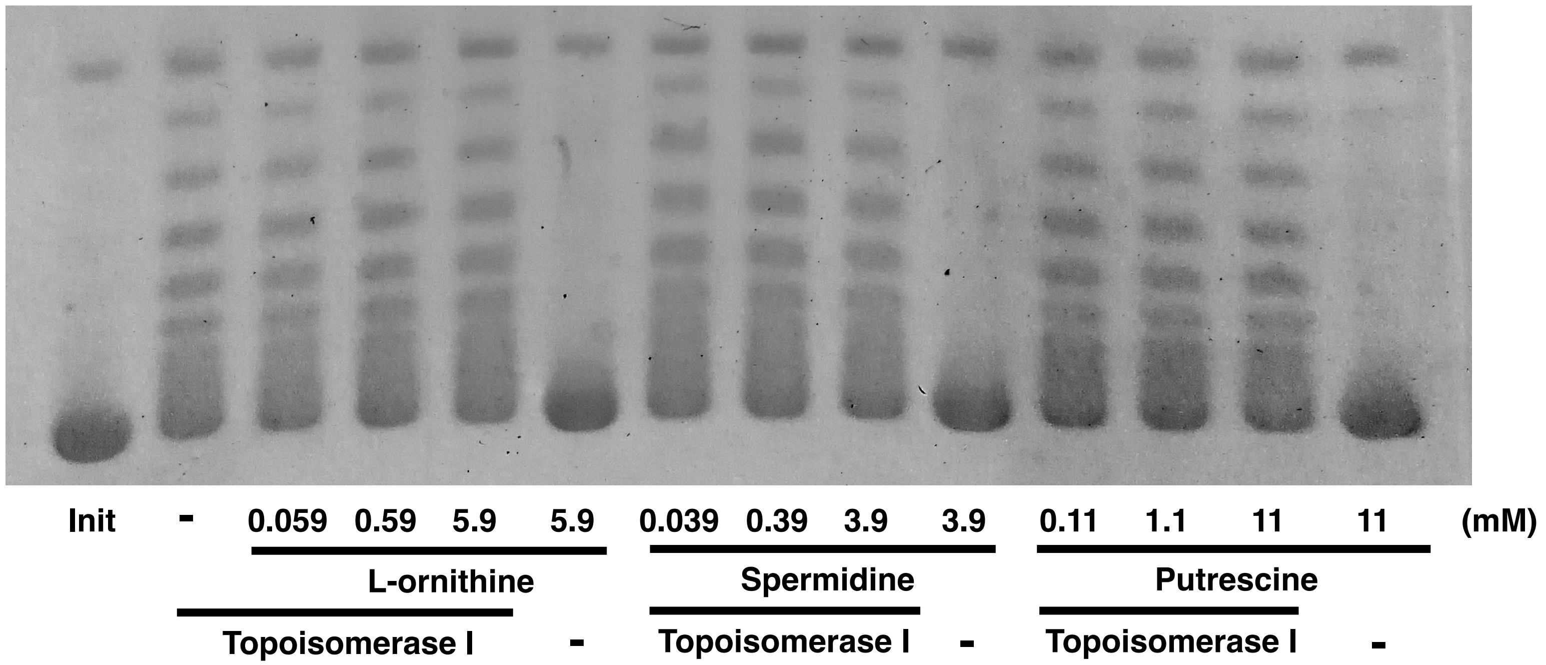

Figure S2: ***In vitro*** **effect of putrescine, spermidine and ornithine on topoisomerase I**
Assays were conducted as described in Methods using the indicated amounts of the listed metabolites.
