# Supplementary material for: DNA supercoiling differences in bacteria result from disparate DNA gyrase activation by polyamines
Source: PLoS Genet. 2020 Oct 30;16(10):e1009085. doi: 10.1371/journal.pgen.1009085 (PMC7598504; doi:10.1371/journal.pgen.1009085)
Supplement: S3 Fig — Intracellular ATP concentrations of wild-type S. Typhimurium (14028s) were measured using the Bac-Titer Glo assay. *: p<0.05 (Student’s t-test, n = 3) (PDF) [file pgen.1009085.s003.pdf]

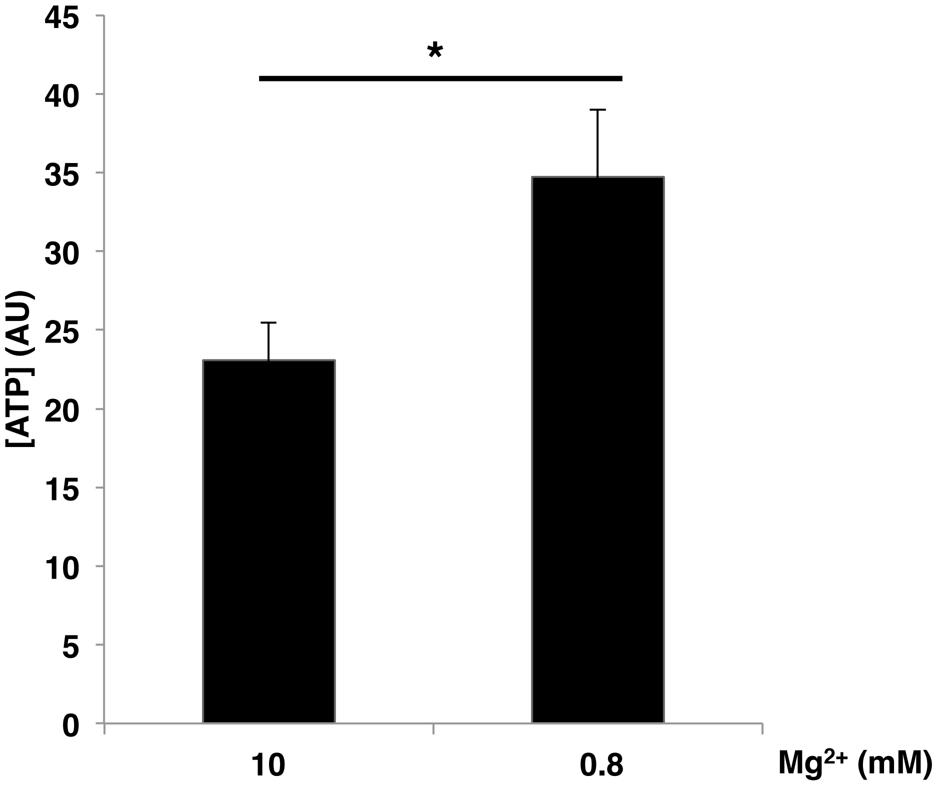

Figure S3: ATP amounts vary little in response to excess Mg^{2+}

Intracellular ATP concentrations of wild-type *S.* Typhimurium (14028s) were measured using the Bac-Titer Glo assay. *: $p<0.05$ (Student's t-test, n=3)
