# Supplementary material for: DNA supercoiling differences in bacteria result from disparate DNA gyrase activation by polyamines
Source: PLoS Genet. 2020 Oct 30;16(10):e1009085. doi: 10.1371/journal.pgen.1009085 (PMC7598504; doi:10.1371/journal.pgen.1009085)
Supplement: S4 Fig — Agmatinase and ornithine decarboxylase activities were assayed in crude extracts from wild-type S. Typhimurium (14028s). ns: not significant (Student’s t-test, n = 3) (PDF) [file pgen.1009085.s004.pdf]

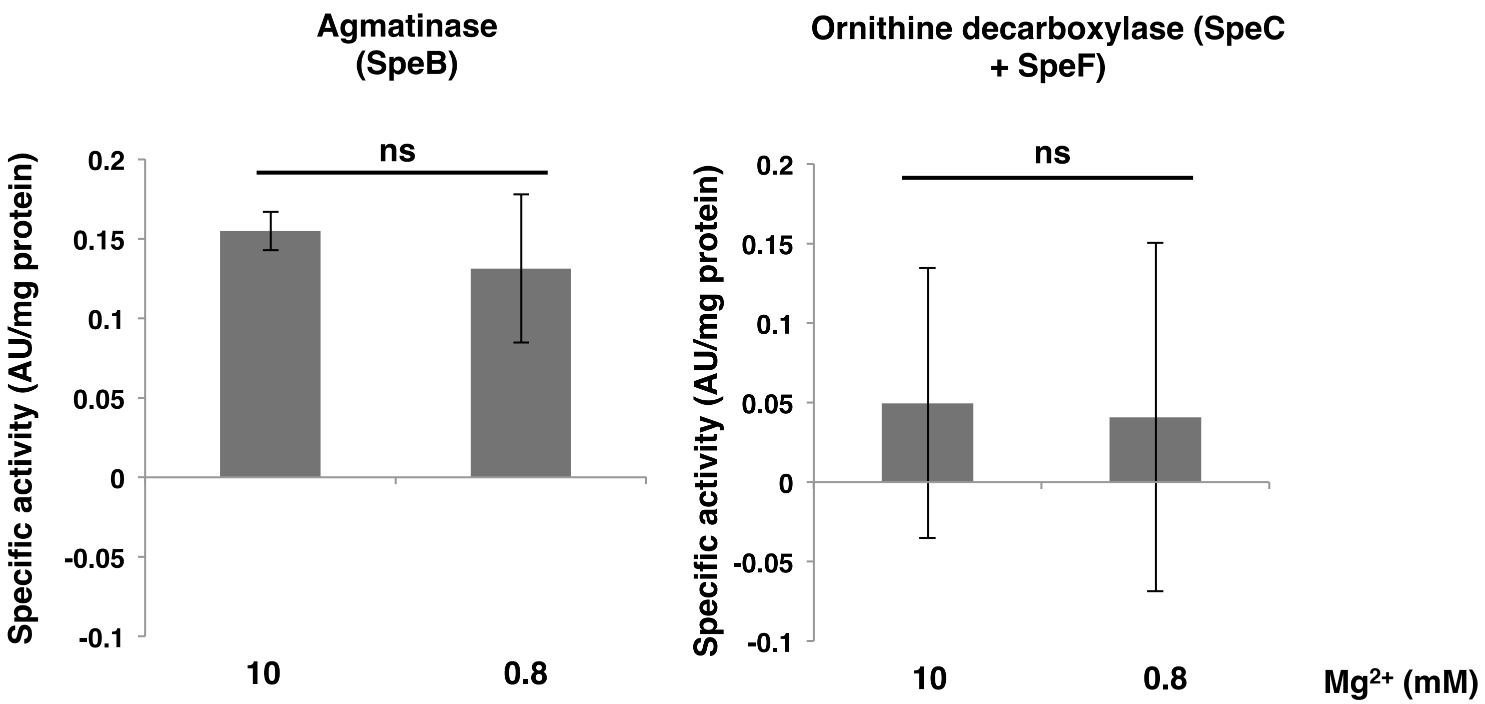

Figure S4: Activity of putrescine biosynthetic enzymes in crude S. Typhimurium extracts
Agmatinase and ornithine decarboxylase activities were assayed in crude extracts from wild-type *S.* Typhimurium (14028s). ns: not significant (Student's t-test, n=3)
