# Supplementary material for: DNA supercoiling differences in bacteria result from disparate DNA gyrase activation by polyamines
Source: PLoS Genet. 2020 Oct 30;16(10):e1009085. doi: 10.1371/journal.pgen.1009085 (PMC7598504; doi:10.1371/journal.pgen.1009085)
Supplement: S5 Fig — Expression of the presented S. Typhimurium genes (in FPKM) was plotted as a function of DNA supercoiling (data taken from GEO entry GSE137586). Open circles represent independent data points, and lines represent linear regressions. (PDF) [file pgen.1009085.s005.pdf]

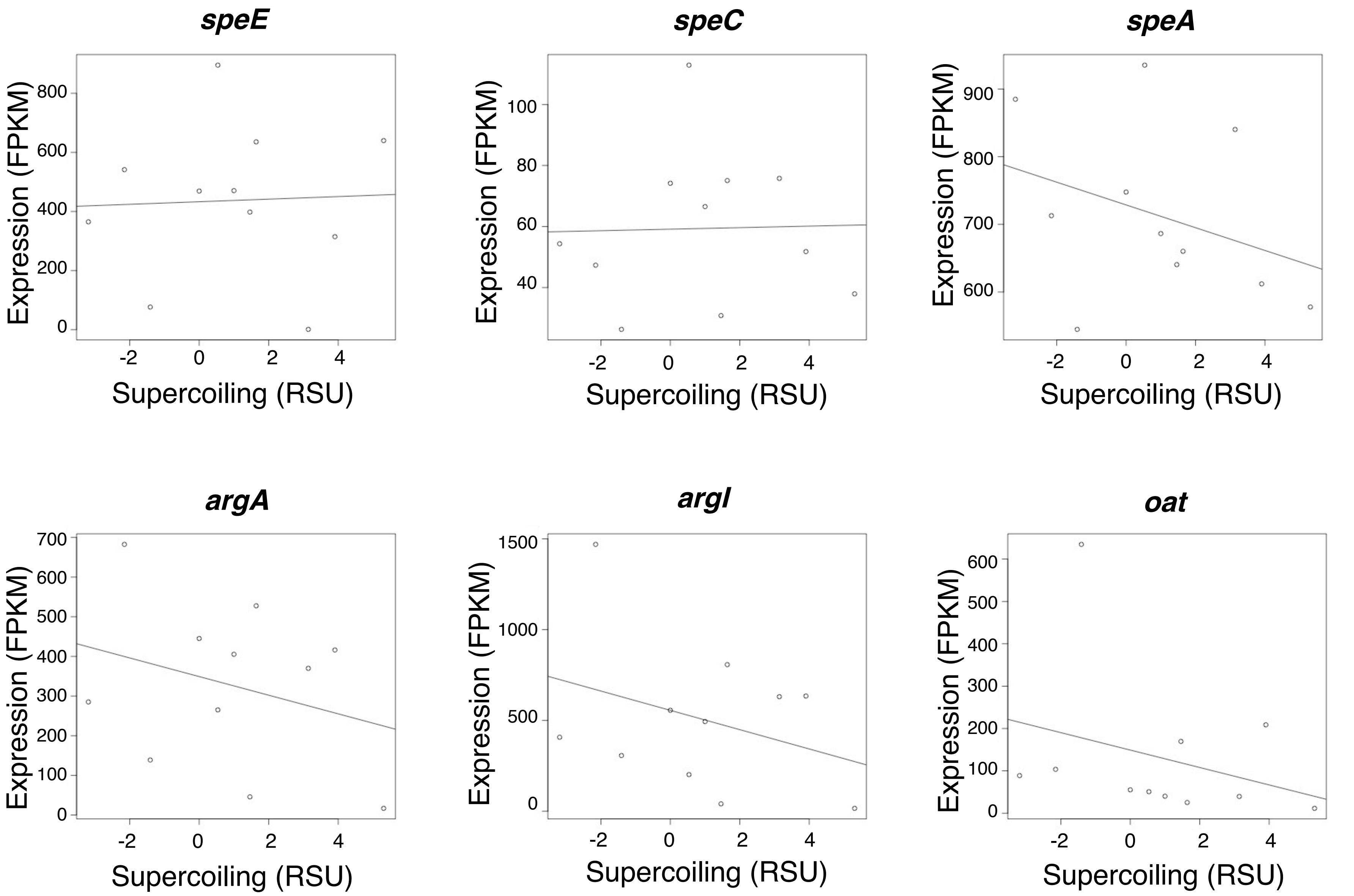

Figure S5: Expression of key putrescine-related genes is not dependent on DNA supercoiling.
Expression of the presented *S.* Typhimurium genes (in FPKM) was plotted as a function of DNA supercoiling (data taken from GEO entry GSE137586). Open circles represent independent data points, and lines represent linear regressions.
