# Supplementary material for: DNA supercoiling differences in bacteria result from disparate DNA gyrase activation by polyamines
Source: PLoS Genet. 2020 Oct 30;16(10):e1009085. doi: 10.1371/journal.pgen.1009085 (PMC7598504; doi:10.1371/journal.pgen.1009085)
Supplement: S6 Fig — Note that OAT is unrelated to the bacterial Oat and catalyzes a different reaction. (PDF) [file pgen.1009085.s006.pdf]

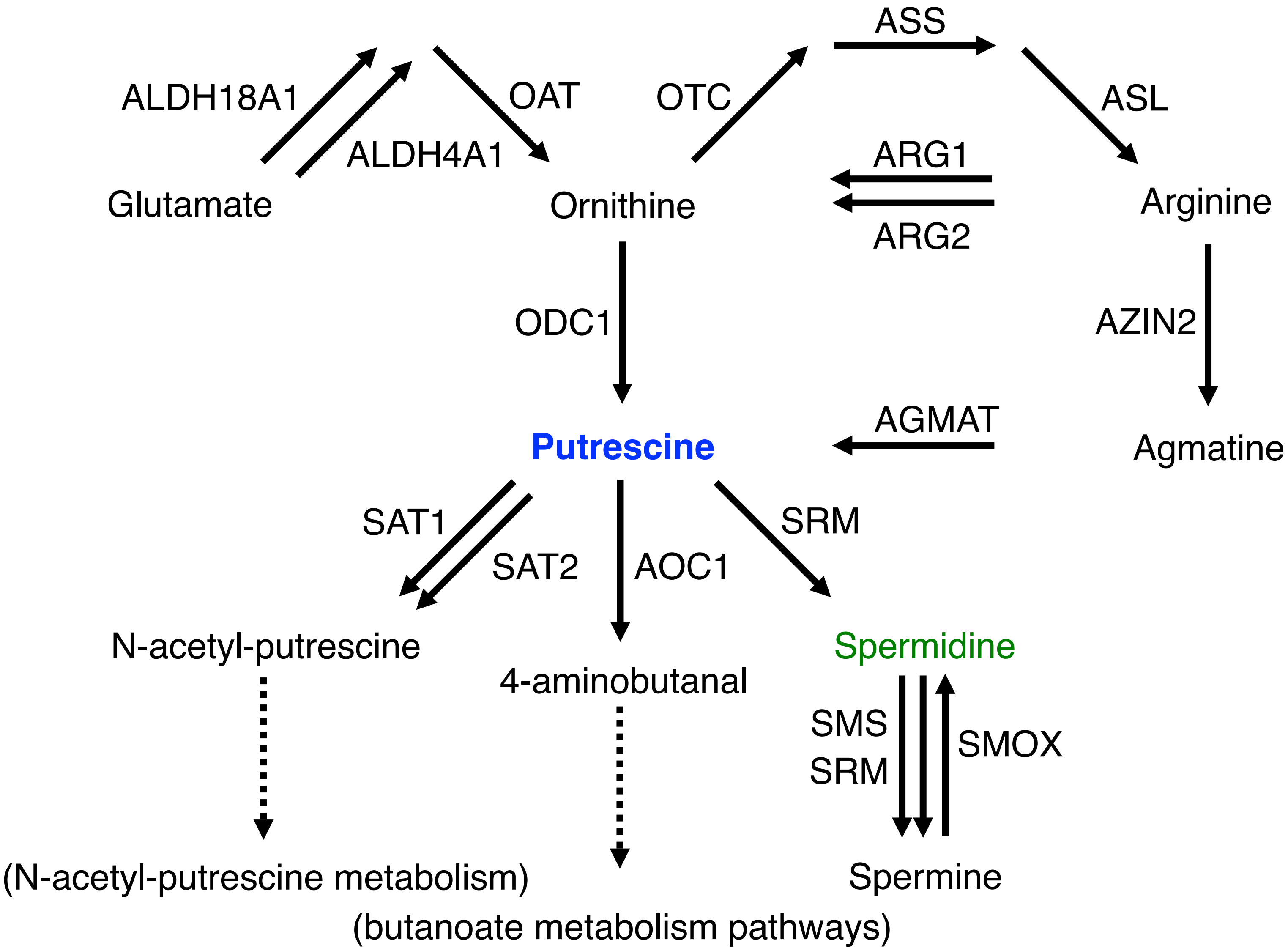

Figure S6: The putrescine biosynthetic and degradative pathways in *H. sapiens*
Note that OAT is unrelated to the bacterial Oat and catalyzes a different reaction.
