# Supplementary material for: DNA supercoiling differences in bacteria result from disparate DNA gyrase activation by polyamines
Source: PLoS Genet. 2020 Oct 30;16(10):e1009085. doi: 10.1371/journal.pgen.1009085 (PMC7598504; doi:10.1371/journal.pgen.1009085)
Supplement: S1 Table — (PDF) [file pgen.1009085.s007.pdf]

Table S1: Strains, plasmids and primers used in this study

Strains	Description	Ref
S. enterica		
14028s	Wild-type *S. enterica* serovar Typhimurium	[1]
AAD46	14028s *speE::cat*	This work
JY979	14028s Δ*oat*	J . Yeom, personal communication
AAD58	14028s *speE::cat* Δ*oat*	This work
EG6501	14028s *sapABCDF::kan*	[2]
AAD61	14028s *argE::kan*	This work
AAD62	14028s *speB::cat*	This work
AAD65	14028s *ΔspeB*	This work
AAD181	14028s *speE::cat* Δ*oat argA::kan*	This work
AAD212	14028s *ΔspeB argE::kan*	This work
E. coli		
MG1655	Wild-type *E. coli* K12, F- λ-	
AAD85	MG1655 *speE::kan*	This work

AAD86	MG1655 *oat::cat*		This work
AAD87	MG1655 *speC::kan*		This work
AAD88	MG1655 *Δoat*		This work
AAD89	MG1655 *speB::cat*		This work
AAD90	MG1655 *speF::cat*		This work
AAD91	MG1655 *puuA::cat*		This work
AAD92	MG1655 *ΔspeB*		This work
AAD95	MG1655 *ΔspeB speC::kan speF::cat*		This work
AAD96	MG1655 *Δoat speE::kan puuA::cat*		This work
Plasmids	Description		Ref
pJV	Standard vector for DNA supercoiling measurements		[3]
pKD3	Template vector for λred recombination, Cm^R		[4]
pKD4	Template vector for λred recombination, Kan^R		[4]
pSIM6	λred plasmid, heat-inducible		[5]
pCP20	FLP recombinase plasmid, heat-inducible		[4]
Primers	Sequence	Target	Ref

16651	ATTATGTTGCGCCCTTTTTTTACGG<br>GTGTTAACAAAGGAGGTATCAACC<br>CGTGTAGGCTGGAGCTGCTTC	*speE*,<br>*S*. Typhimurium	This work
16652	AGATTATTAAAGCCATGCAGTTTC<br>AGTTTTTTCAATTTCTTATCTTCTCC<br>CATATGAATATCCTCCTTAG		This work
16698	ATAATTAATGCATGAATATTGATA<br>CTATCATGACCTGAGGTGTGTCAA<br>CAGTGTAGGCTGGAGCTGCTTC	*argE*,<br>*S*. Typhimurium	This work
16699	CTACAAGACGGGGTAGGCCCGGTA<br>AACATCACGTCACCGGGCAATATG<br>CACATATGAATATCCTCCTTAG		This work
16702	CTAACGACGCGGGAGGGTTTTTTTA<br>TATTGACTAAGAAGAGGTTTTTGCC<br>GTGTAGGCTGGAGCTGCTTC	*speB*,<br>*S*. Typhimurium	This work
16703	CATCAATCAGCTGTAGGCCGGATA<br>AGCGCAGCGCCATCCGGCAAAAC<br>AACCATATGAATATCCTCCTTAG		This work
16832	AACGACGCGGAAGGGTTTTTTTAT<br>ATCGACTTTGTAATAGGAGTCCATC<br>CGTGTAGGCTGGAGCTGCTTC	*speB*, *E. coli*	This work

16833	CGCATCCGACATTAATGGCACGTTT<br>TACCCGTGCGCATCGCATCTGGTGC<br>CATATGAATATCCTCCTTAG		This work
16834	ATTATGTTGCGCCCTTTTTTTACGG<br>GTGTTAACAAAGGAGGTATCAACC<br>CGTGTAGGCTGGAGCTGCTTC	*speE, E. coli*	This work
16835	AGATTATTAAAGCCATGCAGTTTC<br>AGTTTTTTCAATTTCTTATCTTCTCC<br>CATATGAATATCCTCCTTAG		This work
16836	TCATTCGAGAAATTGAGGACCTGC<br>TATTACCTAAAATAAAGAGATGAA<br>AAGTGTAGGCTGGAGCTGCTTC	*speF, E. coli*	This work
16837	CGTCAGTATGGTTAACTGAACGAC<br>GCCCATTTTGTTCGATTTAGCCTGA<br>CCATATGAATATCCTCCTTAG		This work
16838	GTTTTCCACCTTGTCCGGTATTCTTA<br>CTTCCCCGAAACGGGTTTGCGCTTG<br>TGTAGGCTGGAGCTGCTTC	*speC, E. coli*	This work
16839	GGTCGCCAGAAGGTGACCCGTTTTT<br>TTTATTCttaCTTCAACACATAACCC<br>ATATGAATATCCTCCTTAG		This work
16840	TGCAATACTTAAATCGGTATCATGT<br>GATACGCGAGCCTCCGGAGCATAT	*oat, E. coli*	This work

	TGTGTAGGCTGGAGCTGCTTC		
16841	GTCGTATAAAAAGATCGGATGGCG ACGTCGTATCGCCATCCGATTTGAT ACATATGAATATCCTCCTTAG		This work
16842	ATATTTTACGCTTTGATAACGAGCG GAAAACAAACCAAAGGCGAAGAA TCGTGTAGGCTGGAGCTGCTTC	*puuA, E. coli*	This work
16843	ATGTCAGGCCTGGCTCCGCTCAGG CCGATGAAACAACCCCGCAAGGG GTACATATGAATATCCTCCTTAG		This work

References

1. Fields PI, Groisman EA, Heffron F. A Salmonella locus that controls resistance to microbicidal proteins from phagocytic cells. Science. 1989;243: 1059–1062. doi:10.1126/science.2646710

2. Parra-Lopez C, Baer MT, Groisman EA. Molecular genetic analysis of a locus required for resistance to antimicrobial peptides in Salmonella typhimurium. EMBO J. 1993;12: 4053–4062.

3. Duprey A, Muskhelishvili G, Reverchon S, Nasser W. Temporal control of Dickeya dadantii main virulence gene expression by growth phase-dependent alteration of regulatory nucleoprotein complexes. Biochim Biophys Acta. 2016;1859: 1470–1480. doi:10.1016/j.bbagrm.2016.08.001

4. Datsenko KA, Wanner BL. One-step inactivation of chromosomal genes in Escherichia coli K-12 using PCR products. Proc Natl Acad Sci. 2000;97: 6640–6645. doi:10.1073/pnas.120163297

5. Datta S, Costantino N, Court DL. A set of recombineering plasmids for gram-negative bacteria. Gene. 2006;379: 109–115. doi:10.1016/j.gene.2006.04.018
